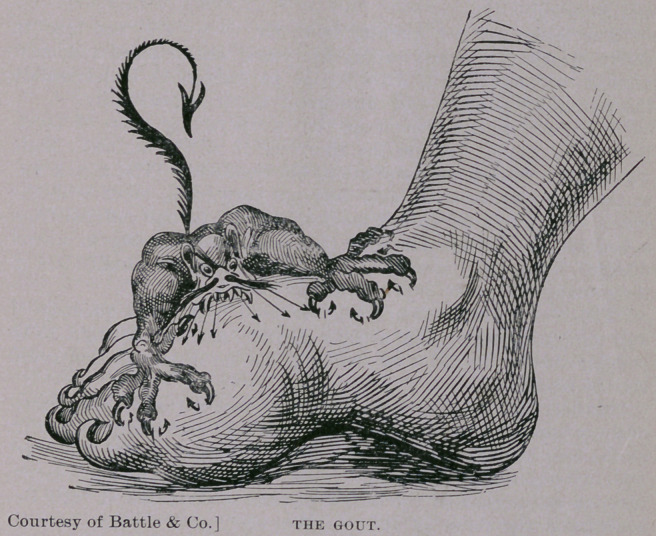# Uric Acid Toxaemia

**Published:** 1900-09

**Authors:** Arch Dixon

**Affiliations:** Henderson, Ky., Ex-President of the Mississippi Valley Medical Association, ex-president Kentucky State Medical Society; Member of the Kentucky State Board of Health, Etc, Etc.


### Uric Acid Toxaemia.

---

ARCH DIXON, M. D., HENDERSON, KY.,
Ex-President of the Mississippi Valley Medical Association, ex-president Kentucky State Medical Society; Member of the Kentucky State Board of Health, etc, etc.

That "necessity is the mother of prevention" is no less true in medicine than in other ways. A distinguished physician living in the East, big of brain and heart, a leader in society work, both literary and social, a "chutmuck," a "friendly Indian," and a member of the "gang," from some cause, upon the discussion of which it is not necessary to enter here, found himself the unwilling victim of increasing uric acid trouble. The attacks gradually became more violent and increased in frequency until life to him because almost a burden. He was compelled to restrict himself in many ways, both in his business and social duties, and he had to turn with serious though to some method of exorcising this demon which had entered into him. How he suffered, only those who have had a similar experience can at all appreciate. I remember once, it was at the Nashville meeting of the American Medical Association; after the business of the day, there was a meeting of the "gang" called together by the chairman of the committee on "Nutrition and Stimulation." Mathews was there and Love and Owen, of blessed memory, and the peerless Palmer, who since "has passed the gates of sorrow through," and McMurtry, Grant and Charley Reed and other royal fellows. There had been a flood of eloquence and an avalanche of wit and the gentleman from the East was much in evidence. An hour later he writhed in agony, the victim of an explosive attack of nephritic colic. Six hours later he was on his way back to the East. His frame of mind was such that he could not exclaim, "This is a very beautiful world and I'm glad I'm living," but the contrary. Returning home he consulted the best men in the profession, among them his particular friends, Price, Wyeth, McBurney and Henry O. Marcy. There was no difference of opinion as to his case. Each and every one pronounced it stone in the kidney, for the relief of which an operation must be done. What did that mean? It meant the indefinite relinquishment of a practice already crippled by continued attacks of indisposition, the leaving of home and family, the subjection of himself to the surgeon's knife, with perhaps the result at best, of a long and tedious convalescence. The contemplation of this gave him pause, and he seriously considered if "the game were worth the candle." But something must be done, and quickly, too, for in addition to this kidney trouble, gout, that torturing devil so aptly portrayed in the accompanying cut, seized upon him. Look upon the picture and it goes without the saying that a further description of his condition is unnecessary. And so this man, selfishly if you will, set the machinery of his great brain to work to discover something to cure himself. It is useless to say that he had tried all remedies known to the profession, holding fast only to those which seemed good. Among all these the lithias gave promise of the best results, and along these lines he began a most active research. Assisted by an able German chemist a series of experiments were kept up for four long years, until finally a laxative salt of lithia was evolved, which, after numerous tests, was found to be an active stimulant to all the emunctories, and since gout is rarely, if ever, due to excessive formation of uric acid in the blood, but always to retention, or failure of excretion, the discovery of this remedy came to this man as a life saver and a blessing indeed. Its action upon him he describes as little short of marvelous, transforming him, as it were, into a new man. Not content to form a verdict from its action in his own particular case, he distributed a sufficient quantity for trial in similar cases among his numerous friends in the profession, telling them of the great things that it had done for him. The reports from these were awaited with great anxiety,

Courtesy of Battle & Co.] THE GOUT.

but at last they came and he could well exclaim "Eureka!" for the results upon others were the same as upon himself. Urged by his friends he determined to give to others the benefit of this great blessing which had come to him, following the injunction:

Have you had a blessing shown?
Pass it on.
'Twas not given for you alone—
Pass it on.
Let it travel down the years,
Let it wipe another's tears.
Till in heaven the deed appears,
Pass it on.

In the spring of 1899 I received several bottles of this lithia salt, now called Thialion, with the request that I give it a trial, and if it proved satisfactory to so report. Being a lithæmic myself, and having run the scale of all remedies recommended for this trouble with little benefit, I was extremely skeptical of its efficacy. However, I concluded to use it in a few very obstinate cases, which had refused altogether to yield to other treatment, or had been very slightly benefited thereby. The results obtained were in the nature of a very agreeable surprise. First of all, I cannot do better than give a report of my own case as taken from a paper, "Some Observations of Lithæmia," by my son, Dr. Arch Dixon, Jr., published in the May number of *The Louisville Monthly Journal of Medicine and Surgery.*

"The latter part of August, 1899, Dr. ——, who has been in active practice for nearly twenty-five years, was attacked suddenly, after a moderate lunch, with vertigo so decided as to necessitate the recumbent posture, and cause great alarm to his family.

"There was no actual syncope, but a distressing sense of faintness, from which, however, he recovered in a few minutes; there was neither nausea nor palpitation, but headache. The attack was at the time attributed to lager beer not very fresh, taken with the lunch. In early life, while a medical student, he had suffered from a bad attack of dyspepsia with palpitation culminating in mitral disease. Occasional attacks of vertigo occurred, but usually late in the evening and after days of unusual fatigue.

"These were always temporarily relieved by a small quantity of any mild stimulant. The attacks were at one time thought to be possibly due to his habit of smoking, but no direct relation could ever be traced.

"Matters had now assumed so grave an aspect that he began seriously to study his own case as he would have been compelled to do in the case of any other patient. First the condition of the heart was investigated as a possible cause, but competent examination revealed no increase of the mitral disease, no evidence of fatty degeneration, the pulse in fullness, frequency and rhythm normal, neither palpitation nor dyspnœa, only an occasional intermission of the pulse. No evidence whatever of any organic disorder. The renal function was apparently perfect; the urine of proper specific gravity, although there was a tendency to abnormal acidity. In the absence of any deposit or other symptom, the urine was only roughly tested at any time, until a severe attack of lumbago accompanied by general myalgia and intense headache compelled a more accurate examination. The digestion was bad; the bowels as always during life, regular with the exceptions noted hereafter; the urine was found to be loaded with uric acid. These attacks have never been accompanied by fever nor by any severe disturbance of the general health, but always by extreme irritability, nervousness and impatience, with more or less torpor of the bowels. The appetite, even in the worst of these, was always good enough, if not too good. A more careful course of diet was at once instituted.

"The amount of nitrogenous and carbonaceous food was greatly reduced, and all stimulants and malt liquors, always in daily, but never in excessive use were discarded entirely, smoking was interdicted.

"As medicines, a full dose of Thialion was given before each meal, and an active dose of concentrated French lick water on rising each morning, these producing one full liquid evacuation daily. The effect of this course was very decided. It was continued with hardly an intermission for four months, though on several occasions, when too much animal food, a glass or two of wine or whiskey were indulged in, the warnings were unmistakable. At the end of this period the tinnitus was hardly noticeable, the vertigo entirely gone, and the gouty pains a thing of the past. His health has been more vigorous than ever, but only at the price of constant watchfulness, for any attempt at the indulgence of the table, either at once or with the lapse of two or three days, brings its penalty in myalgic pains, with headache, tinnitus, or vertigo one or all. The only wines that seem to cause no trouble are a thin table sherry and dry champagne."

I make no apology for giving the case at some length, and I consider it to be a good illustration of a certain class of lithæmic cases, and typical of the nervous and gouty complications, while remarkably free from those renal and gastric symptoms which more generally accompany and obscure the diagnosis, for, as will be noticed, there were none of the ordinary symptoms to call attention to what was undoubtedly the true source of the difficulty, the imperfect assimilation of the ingesta. That vertigo and tinnitus as well as other obscure and intractable complaints, especially those of the skin and mucous membranes, may often be traced to the lithuric condition, whether it be designated as lithæmia or suppresed gout, there can be no doubt.

Case 2. Mr. A. J. C., age 67, farmer, weight 180, was referred to me by another physician. Mr. C. had led an active life, was a good eater, but moderate drinker. For more than two years he had been suffering with lumbago, headache and what he described as "spells," in which he would for the moment lose consciousness. These attacks of vertigo would come on suddenly and without periodicity. He was also much constipated, was low spirited, being convinced that he was the victim of some incurable malady. His digestion was bad, complexion sallow, tongue coated. His urine was scanty. Specific gravity 1.030, reaction strongly acid, with a trace of albumin, dark red, with a large brick dust deposit. Microscopic examination revealed uric acid crystals, in large numbers, with a corresponding deficiency in amorphous urates. He was put upon teaspoonful doses of Thialion in a glass of hot water three times daily before meals. He was also instructed as to diet and told to take a hot bath every night just before retiring and to report in a week or ten days. In a week Mr. C. came into my office and reported that he was much better. The same treatment ordered except that the Thialion was to be taken only once a day, an hour before breakfast.

In three weeks he came in and said, "Doctor, I am a new man, my friends ask me what I have been doing to myself, I look so much better they hardly know me." Mr. C. is a new man, his aches and ailments have all disappeared and with the exception that he still has an occasional mild attack of vertigo, his health is excellent. I could cite numbers of such cases.

One of the most striking and convincing effects of the virtues of Thialion is in the treatment of asthma. There can be no question, as Haig says, that uric acid produces high arterial tension and that the contraction of the arterioles varies directly with the amount that is circulating in the blood. The way then to cure asthma is to cleanse the blood of uric acid and to keep it clean; this Thialion will do. Mrs. D., aged 65, had suffered for years with frequent attacks of asthma. She had been under the treatment of several physicians, who had exhausted all the remedies known to pharmacy with but temporary relief, and this being afforded only by morphia hypodermically administered. Her breathing was at all times labored, but during the paroxysm the dyspnœa was painful to see, and sleep was out of the question. Examination of the chest revealed bronchial catarrh and emphysema. The slightest change in atmospheric conditions aggravated all symptoms and she looked forward with dread to those changes which brought about humidity. She became a regular barometer and the signal service man was not in it with her in foretelling the changes in the weather. Thialion was given her in the usual doses with the addition, now and again, of a teaspoonful of elix paraldehyde at bed time. It has now been four months since she began the treatment and with the exception of the remaining emphysema she is practically a well woman and is correspondingly grateful.

It is useless to multiply cases. The production of uric acid is an interesting and as yet an unsettled problem. Whether it be by synthesis of the ammonium salts with lactic acid in the liver, or by decomposition of nuclein, or by the kidney synthetizing urea with glycine, the fact remains that it is there and the prime object is to get rid of it. The pains in the muscles and fibrous tissues and even in joints, that often appear after exposure to cold and like influences, though called rheumatic are often not rheumatic at all, but are manifestations of metabolic disturbances and due to alterations in the fluids of the body, in consequence of which certain substances are thrown out of solution and act as irritants, generally or locally. These cases derive no benefit from the salicylates, but are certainly and surely relieved by Thialion. Lest we forget, let us remember that all uric acid troubles, and they are legion, are due to disturbed metabolism and lessened excretion, and that by complying with certain rules of diet, the best of which are those laid down by Dr. Henry S. Pole, of Hot Springs, Va., and by giving such remedies as increase excretion and restore metabolic equilibrium, and the best of these in so far as my experience goes is Thialion, we can restore our patients, if not to perfect health, to that condition in which he may now and again exclaim, "This is a very beautiful world and I'm glad I'm living."

## Figures and Tables

**Figure f1:**